# Smoking cessation and harm reduction: a systematic overview of ongoing, randomized controlled trials

**DOI:** 10.1186/s12888-024-06342-6

**Published:** 2024-12-17

**Authors:** Mara Derissen, Sebastian Scheliga, Benjamin Clemens, Delia Leiding, Knut Kröger, Hinrich Böhner, Ute Habel

**Affiliations:** 1 Department of Psychiatry, Psychotherapy and Psychosomatics, Faculty of Medicine, RWTH Aachen University, Pauwelsstraße 30, Aachen, 52074 Germany; 2 Institute of Neuroscience and Medicine, JARA-Institute Brain Structure Function Relationship, Research Center Jülich, Jülich, Germany; 3 Clinic for Angiology, Helios Hospital Krefeld, Krefeld, Germany; 4 Department of General and Visceral Surgery, Catholic Hospital Dortmund West, Dortmund, Germany

**Keywords:** Smoking cessation, Harm reduction, Randomized-controlled-trials, Repository

## Abstract

**Background:**

Smoking remains one of the major public health threats, necessitating substantial scientific and societal interest in further developing and implementing systematic, smoking cessation trials. This review examines ongoing randomized controlled trials (RCTs) on smoking cessation and harm reduction, focusing on adherence to German S3 guidelines for tobacco dependence and identifying areas needing further research and neglected aspects in the implementation of treatment guidelines for tobacco dependence.

**Methods:**

A systematic search was conducted on the International Clinical Trials Registry Platform, comprising multiple trial registries worldwide, to identify ongoing RCTs focusing on smoking cessation and harm reduction. Utilizing the PICOS-scheme, we focused on trials targeting the general population, with biochemical verification, psychological counseling, telemedicine, and nicotine replacement therapy /smoking cessation medication or electronic nicotine delivery systems. Exclusion criteria included trials marked as "completed," "terminated," "unknown," or "withdrawn.".

**Results:**

The review identified 30 ongoing RCTs, with a majority located in North America. A significant number of trials focus on socioeconomically disadvantaged or uninsured populations, while few address cancer survivors or individuals with smoking-related diseases. Nicotine replacement therapy or smoking cessation medication is consistently used across trials, but with varying regimens. Psychotherapeutic interventions are employed in 22 trials, with motivational interviewing being the most common method. Only four trials utilize electronic nicotine delivery systems as a harm reduction strategy. The lack of standardized reporting in trial registries was a significant barrier to synthesizing and categorizing information. Geographic representation is predominantly in North America, suggesting a need for more diverse trial locations.

**Conclusions:**

There is a critical need for more RCTs involving electronic nicotine delivery systems and tailored psychotherapeutic interventions. Expanding trial locations beyond North America and standardizing trial reporting could enhance the global applicability of smoking cessation strategies. Future research should focus on the long-term risks and benefits of electronic nicotine delivery systems, particularly in high-risk populations. This approach will aid in developing more effective and culturally relevant smoking cessation guidelines.

**Supplementary Information:**

The online version contains supplementary material available at 10.1186/s12888-024-06342-6.

## Background

The need for dedicated and specialized help to quit tobacco use is well-documented: without systematic cessation programs or individual support structures, only 4% of cessation attempts are successful [1]. Professional and systematic cessation support more than doubles a user's chance of successfully quitting [1]. In the last four decades, considerable effort has been devoted to investigating systematic attempts at smoking cessation. Many RCTs have been published, focusing either on the general population or, more specifically, high-risk populations [2, 3]. This research is particularly relevant for mental health professionals, given the disproportionately high rates of smoking among psychiatric patients and their unique opportunities for intervention. In the last four decades, considerable effort was devoted to investigating systematic attempts at smoking cessation. A comprehensive overview of these studies reveals some consistent findings. Physicians addressing people who smoke through brief but structured consultations is far more effective than no advice [4]; brief counseling by physicians was significantly superior to either no counseling or usual care (RR = 1.66). Motivational Interviewing (MI), a behavioral intervention and counseling method that explores and resolves ambivalence and facilitates change, has been shown to moderately increase quit rates [5]. However, a more recent meta-analysis by Lindson and colleagues [6] questioned the effectiveness of MI. Furthermore, the effectiveness of psychotherapeutic methods seems strongly dependent on the individual characteristics of people who smoke, including gender, age, and somatic or psychiatric comorbidities. Additionally, a combination of cognitive behavioral therapy (CBT) and pharmacological smoking cessation aids has proven effective. Combining bupropion and CBT (OR = 1.83) or varenicline with CBT (OR = 2.96) has shown greater effectiveness [7]. Recently, internet-based intervention programs have become popular in smoking cessation trials. However, internet-based interventions that involve no direct contact or physical meetings with psychologists or physicians are only slightly superior to passive control groups receiving usual care or self-help materials (RR = 1.15) [7]. Nicotine replacement therapy (NRT) is the most common form of smoking cessation support, as it can satisfy addiction and prevent tobacco use. NRT supplies people who smoke with nicotine – in the form of gum, patches, or sprays – without the additional harmful chemicals in tobacco. NRT is intended to ameliorate and reduce physical withdrawal symptoms so individuals can focus on the psychological aspects of quitting. A recent Cochrane meta-analysis evaluated 14 RCTs on the benefits of NRT for smoking cessation [8]. Compared to a placebo, NRT significantly increased the likelihood that people who smoke and are unwilling to quit reduce daily cigarette use by at least half (RR = 1.75). NRT also significantly enhanced the likelihood of subsequent smoking cessation (RR = 1.87). In addition to NRT, another type of pharmacological treatment for smoking cessation includes medications such as varenicline, cytisine, and bupropion, which have proven effective as smoking cessation aids. A recent Cochrane review has demonstrated high-certainty evidence that varenicline and cytisine increase quit rates [9].

Another current and controversial smoking cessation aid, categorized as a harm reduction strategy, is the use of electronic nicotine delivery systems (ENDS). ENDS encompass e-cigarettes, vaping devices used to “vape” a flavored liquid that may or may not contain nicotine, as well as heat-not-burn (HNB) products. HNB products are devices that heat processed tobacco to lower temperatures than combustion, producing an aerosol containing nicotine and other compounds without burning the tobacco. While in e-cigarettes the user activates a battery-powered heating element, the liquid is vaporized, releasing an aerosol that contains various substances. This process reduces the levels of harmful byproducts typically associated with traditional cigarette smoke, such as tar and carbon monoxide, while still delivering nicotine to the user. Compared to cigarette smoking, the advocated advantages of ENDS, including HNB products, mainly comprise lower levels of volatile organic compounds and tobacco-specific nitrosamines [10]. ENDS have been the center of heated debates in recent years. On the one hand, they are deemed an alternative to cigarette smoking, beneficial for people who smoke during quit attempts. On the other hand, multiple concerns arose, mainly regarding the risk to public health and their prevalent use among adolescents and youth. An updated Cochrane review of these harm reduction methods included 40 RCTs. The authors concluded that there is moderate evidence (limited by imprecision) that ENDS with nicotine increase quit rates compared to ENDS without nicotine (RR = 1.94). Comparing ENDS with NRT, there is high evidence favouring ENDS, as quit rates were higher among people randomized to ENDS with nicotine than those randomized to NRTs (RR = 1.63) [11]. Moreover, there was moderate evidence (limited by imprecision) that the rate of adverse events (AEs) was similar (RR = 1.02, 95% CI 0.88 to 1.19), supporting a therapeutic implementation of ENDS as a valuable harm reduction strategy.

The WHO implemented Article 14 of the Framework Convention on Tobacco Control (FCTC) to promote smoking cessation efforts globally, obligating participating parties to implement evidence-based measures and guidelines [12]. One hundred sixty-nine countries are parties to this convention committed to reducing tobacco use and its associated health risks. Treatment guidelines for tobacco use and dependence vary among participating countries, but they generally incorporate evidence-based strategies for both behavioral and pharmacological support in smoking cessation efforts. Continuous evaluations of guideline implementations are necessary to optimize their impact and reduce the global burden of tobacco-related diseases. Germany, being a committed party to Article 14 FCTC, follows the WHO’s recommended guidelines for tobacco dependence [13]. The German S3 Guideline "Smoking and Tobacco Dependence: Screening, Diagnosis, and Treatment" provide a comprehensive framework for tobacco dependence treatment. These evidence-based guidelines recommend a stepped-care approach, tailoring interventions to the individual’s needs and level of tobacco dependence. Those interventions include brief interventions by healthcare providers (i.e., following the five A’s framework: Ask, Advise, Assess, Assist, Arrange), intensive behavioral support (e.g., cognitive-behavioral therapy and motivational interviewing), and pharmacological support, including NRT and cessation medications (e.g., varenicline). Harm reduction efforts are not supported by those guidelines as the long-term health consequences and long-term benefits of ENDS as a smoking cessation aid remain debatable. However, German guidelines promote further efforts to continue investing in ongoing RCTs in harm reduction strategies to elucidate their potential as a smoking cessation aid. In this context, we define harm reduction in alignment with internationally recognized standards, including those of the WHO and the S3 guidelines. Harm reduction refers to switching from combustible cigarettes to less harmful alternatives, such as ENDS or other non-combustible nicotine products. Further, we adhere to this definition by focusing on studies where such transitions were evaluated with the ultimate goal of cessation. As researchers from Germany, we are familiar with the domestic treatment guidelines for tobacco dependence, which align with the WHO’s recommended guidelines. Therefore, we chose to follow the S3 Guideline "Smoking and Tobacco Dependence: Screening, Diagnosis, and Treatment" in our review to provide a standardized framework to evaluate current studies on tobacco dependence treatment globally [13].

The primary aim of this review is to systematically and comprehensively review ongoing smoking cessation and harm reduction trials currently running and enrolling participants internationally. Our objectives are to [1] identify currently ongoing smoking cessation and harm reduction trials that follow the S3 treatment guidelines for tobacco dependence, [2] synthesize and categorize the information provided in the trial protocols, and [3] evaluate the quality of data in trial registries. This focus on ongoing trials, rather than completed studies, allows us to identify emerging trends and gaps that can inform future research priorities and resource allocation. Such an approach will help to create a broad picture of how the scientific field is progressing and which aspects of international treatment guidelines might be understudies and underrepresented. Furthermore, it can provide a unique perspective on current smoking cessation and harm reduction interventions that may not yet be captured by published trial results.

## Methods

### Eligibility criteria

We followed the PICOS-scheme (population, intervention, comparison, outcome, study type) to formulate scientific questions and define inclusion and exclusion criteria according to German S3 guidelines. Regarding the population, our review focuses on individuals who smoke daily, do not have a pre-existing smoking-related disease, and aim to quit to prevent the onset of smoking-related health consequences. While all smokers face health risks, we excluded trials specifically targeting pregnant women as these often involve different motivations and treatment approaches that warrant separate analysis. The intervention must include psychological counseling delivered by a human expert or through telemedicine combined with NRT, smoking cessation medication or ENDS. The primary outcome must include participants’ self-reported smoking status and a biochemically verified smoking abstinence. The study type should be interventional, with at least one follow-up measurement. Furthermore, we aim to include studies with at least one control group for comparison, with treatment arms allocated randomly. Table 1 provides a summary of our defined PICOS criteria.

**Table 1:** Summary of the applied PICO criteria

PICOS strategy	Inclusion criteria	Exclusion criteria
P–Population	Adults (aged 18 +) who smoke	Pregnant women
I–Intervention	Behavioral intervention including psychological counseling delivered by a real human expert or telemedical application and pharmacological (e.g., nicotine replacement therapy or smoking cessation medication) interventions or electronic nicotine delivery systems at the individual or group level	
C–Comparison	No intervention; delayed intervention; treatment-as-usual; active comparator	
O–Outcome	Primary outcome will be biochemically verified smoking abstinence (e.g., exhaled CO), while secondary outcome will be self-reported abstinence (e.g., cigarettes per day, self-reported quitting)	
S-Study type	Interventional; trials must obtain at least one follow-up measurement; must include at least one control croup with randomized allocation	

Detailed summary of the applied PICO criteria applied in the review process

### Search methods

We searched the International Clinical Trials Registry Platform (ICTRP), a clinical trials registry operated by the World Health Organization (WHO). This comprehensive approach ensures coverage of all clinical trials worldwide. The database search was conducted on 8 March 2023. A summary of the clinical trial registries providing data to the ICTRP can be found in Table 2. Our goal was to extract relevant information for ongoing clinical trials for smoking cessation and harm reduction. Detailed information on the literature search strategy is provided in the Supplement. This review was not pre-registered.

**Table 2:** Data providers of the ICTRP

Data Provider
Australian New Zealand Clinical Trials Registry (ANZCTR)
Brazilian Clinical Trials Registry (ReBec)
Registries Central Committee on Research Involving Human Subjects (CCMO)
Chinese Clinical Trial Registry (ChiCTR)
Clinical Research Information Service (CRiS)
ClinicalTrials.gov
Clinical Trials Information System (CTIS), European Medicines Agency
Clinical Trials Registry—India (CTRI)
Cuban Public Registry of Clinical Trials (RPCEC)
EU Clinical Trials Register (EU-CTR), European Medicines Agency
German Clinical Trials Register (DRKS)
Iranian Registry of Clinical Trials (IRCT)
ISRCTN
International Traditional Medicine Clinical Trial Registry
Japan Primary Registries Network (JPRN/jRCT)
Lebanese Clinical Trials Registry (LBCTR)
Pan African Clinical Trial Registry (PACTR)
Peruvian Clinical Trials Registry (REPEC)
Sri Lanka Clinical Trials Registry (SLCTR)
Thai Clinical Trials Register (TCTR)

Summary of data providers of the ICTRP search portal

### Data management

**Fig. 1:**
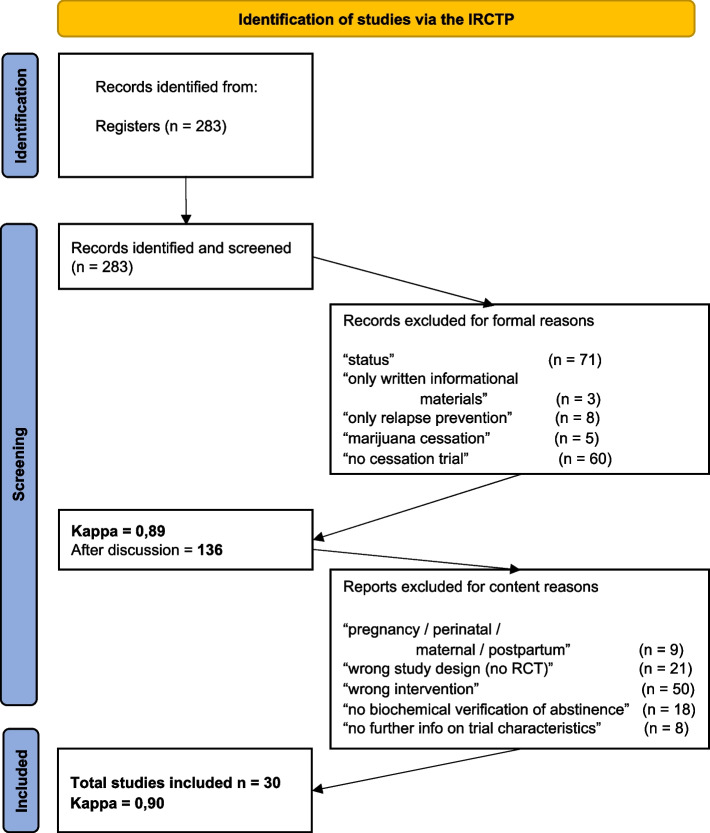
PRISMA flow diagram. PRISMA flow diagram providing details on the number of clinical trials identified and examined

The search results were uploaded and managed in Covidence, a web-based systematic review software program. Covidence allows reviewers to screen the search results online, extract data from relevant clinical trials, perform quality assessments online, and export results. Screening questions based on this review’s inclusion and exclusion criteria were uploaded to Covidence. The PRISMA flow diagram (Fig. 1) details the number of clinical trials identified and examined.

### Study selection process

The initial search identified 283 ongoing smoking cessation studies. Two reviewers independently screened the trial protocols based on the selection criteria. Any discrepancies regarding included or excluded articles were discussed and resolved in a meeting between the reviewers. Reasons for excluding a clinical trial were documented. In the first step, we excluded all studies whose status was "completed," "terminated," "unknown," or "withdrawn," as we were only interested in RCTs that are currently running and enrolling participants. This focus was essential to create a comprehensive overview of ongoing smoking cessation and harm reduction efforts. We excluded trials that only provided written information on smoking cessation to participants, focused solely on relapse prevention, or tested the feasibility or usability of a protocol without defining smoking cessation as a primary outcome. Additionally, we excluded smoking cessation trials related to marijuana consumption. After screening studies based on the above-mentioned formal criteria, the two raters achieved a satisfactory inter-rater agreement of k = 0.89. Consensus was reached for *n* = 136 studies. Furthermore, we screened all 136 eligible studies considering the above-mentioned criteria following the PICOS scheme. We excluded studies examining a specific cohort, such as pregnant women, as smoking cessation is often not the primary goal but an add-on to a primary therapy (e.g., lifestyle intervention) in such cases. The motivation for quitting also differs in those studies, focusing on preventing harm to the unborn child rather than improving individual health outcomes. Therefore, we excluded all RCTs specifically designed for pregnant women to focus on smoking cessation for improving individual health outcomes in the general population. Two raters conducted the screening independently, and conflicts were resolved by consensus or consultation with a third team member. The screening process resulted in a total of 30 ongoing RCTs, with an inter-rater agreement of k = 0.90. The review was conducted without a pre-prepared protocol.

### Data extraction

Data extraction forms were designed and agreed upon by the authors. Data were independently extracted by one research scientist and checked by a second. Discrepancies were identified and resolved through discussion. For each clinical trial, information regarding study characteristics was extracted, including the responsible party, study setting, population and participant characteristics, interventions, comparators, outcome measures, and study design. We aimed to assess the interventions, outcome measures, population characteristics, and the quality of the reported information on the ICTRP.

### Synthesis

Since there was no data on the trial results to conduct a meta-analysis, the trials were analyzed using narrative synthesis.

### Risk of bias

We did not conduct a risk of bias assessment of the ongoing RCTs in this systematic review. The purpose of a risk of bias assessment is to identify bias introduced into the results of an RCT. Since we only included ongoing RCTs in this systematic review, it was impossible to assess possible biases in the results.

## Results

An overview of the 30 RCTs that met all criteria according to our PICOS scheme can be found in Table 1 in the Supplement, comprising relevant categories such as responsible party, setting and trial location, population, intervention components, intervention dosing, and measurement of smoking cessation. A detailed summary of smoking cessation intervention components in the 30 RCTs is illustrated in Table 2 in the Supplement.

### Population

All RCTs are individually randomized and conducted in an outpatient or community setting. The identified trials are conducted in North America, Asia, Europe, Oceania, and Africa, with the majority of trials (*n* = 17) in North America [14–30]. Five trials are carried out in Europe [31–35], and four trials have been initiated in Oceania [36–39]. Furthermore, three trials are from Asia [40, 41], and only one trial is led in Africa [42]. Apart from one trial [43], conducted in North America and Asia, all remaining (*n* = 29) trials are conducted in a single country.

All 30 trials investigate the effects of behavioral smoking cessation interventions in adults who currently smoke. However, 21 of the 30 trials specify high-risk populations in their inclusion criteria, while one includes only dual users of regular cigarettes and ENDS [30]. Two trials only include individuals from African American or Hispanic ethnicities [26, 33]. Cancer survivors or cancer patients who smoke regularly are recruited in two [14, 31] of the 30 trials. Four trials only include socioeconomically disadvantaged, low-income, uninsured individuals [15, 16, 24, 33], while four trials investigate people who smoke who are Human Immunodeficiency Virus (HIV) positive [23, 42, 43]. In contrast, two trials only include patients diagnosed with an acute coronary syndrome [25] or hospitalized with an acute coronary syndrome diagnosis [22], while two trials investigate either people who smoke admitted with a cardiac or pulmonary disease diagnosis [28] or people who smoke suffering from COPD [34]. Another trial requires participants to smoke and currently be scheduled for elective surgery [36], without further specifying the diagnosis. Furthermore, three trials recruit either young veterans with Post-Traumatic Stress Disorder (PTSD) [17], patients with severe opioid addiction [32], or people living with certain chronic conditions [21] who smoke regularly. Finally, one trial involves patients who smoke admitted to several hospital wards (i.e., mental health, orthopedic, plastic surgery, and neurosurgery wards), focusing on the patients admitted to the mental health ward and using patients from the remaining wards as an example for the general population [38].

### Interventions

#### Psychological interventions

Regarding specific characteristics of the employed psychological interventions, 23 [14–17, 19, 21–25, 27–29, 31–36, 38, 40, 42, 43] of the 30 trials implemented different psychological counseling methods by a real human expert with variable content, intensity, frequency of contact, modality of contact, and type of provider. Three trials [15, 16, 27] use financial incentives for treatment engagement and biochemically verified abstinence, while one trial uses integrated financial coaching [24]. General, behavioral, or smoking cessation counseling sessions with a tobacco treatment specialist or a trained counselor are the most common forms of psychological intervention in eleven [15, 16, 32, 34, 35, 42, 43] of the 30 trials. Moreover, one trial [40] utilizes psychoeducational strategies in combination with coaching/counseling. Eleven trials [14, 17, 19, 21, 23, 25, 28, 31, 33, 38] informs on either the content or the underlying psychological frameworks of the psychological intervention in their trial protocols. Conversely, four trials [28, 34, 40, 42] do not provide any further information on the psychological intervention’s content or underlying psychological frameworks. Motivational interviewing is the psychological counseling method of choice in three trials [31, 33, 38]. Two trial protocols [29, 31] specify the counseling method used as cognitive behavioral therapy-based intervention. Furthermore, implemented counseling strategies and psychotherapeutic interventions in the remaining trials (*n* = 8) are Mindfulness-based Addiction Training (*n* = 1) [19], Mindfulness Training (*n* = 1) [14], Managed Problem Solving (MAPS), adherence intervention (*n* = 1) [23], Hypnotherapy (*n* = 1) [31], Acceptance and Commitment Therapy model (*n* = 1) [40], Behavioral Activation based mood management (*n* = 1) [25], strategies based on the Social Cognitive Theory (*n* = 1) [21], and PTSD-informed smoking cessation intervention (*n* = 1) [17].

The duration and frequency of the psychological intervention varies greatly between trials, and some study protocols do not specify the frequency and duration of these interventions. Five trials [14, 19, 32, 38, 43] deliver it weekly or biweekly, whereas the exact contingency of sessions (i.e., without a defined frequency of contact) is stated in nine protocols [15, 17, 21, 23–25, 31, 34, 42]. In those nine trials, the amount of delivered sessions varies from one session to more than eight, with five trials [15, 17, 23, 31, 43] offering less than eight sessions. In addition, four trials [21, 29, 34, 42] offer eight or more sessions of psychological intervention. Moreover, the duration of the psychological intervention varies from as little as 10–20 min (*n* = 2) [15, 43] to more than 20 min (*n* = 3) [17, 25, 40] and an hour or more (*n* = 4) [14, 19, 21, 29].

Similarly, the modality of contact varies, as does the specified information in the trial protocols. In seven [15–17, 19, 21, 36, 38] of the 23 trials that implement a psychological intervention delivered by a real human expert, the intervention is solely delivered via telephone calls or videoconferencing. Three trials [29, 31, 40] offer a hybrid format, with sessions being partly in-person and partly delivered via telephone calls, while one trial [14] delivers the psychological intervention solely in person. Twelve trials [22, 24, 27–29, 31–33, 35, 40, 42, 43] do not state any details on the delivery method of their psychological intervention.

Few trial (*n* = 10) protocols provide details on the type of provider. Eight types of providers are mentioned (i.e., tobacco treatment counselor [28], trained counselor [40, 43], Motivational interviewer [38], psychotherapist/therapist [14, 31], hypnotherapist [31], clinician [27], medical doctor and nurse [33, 34]). Please see the Supplement for a table displaying a detailed summary of the psychological interventions provided in all 30 RCTs.

#### Mobile device-based interventions

Seventeen [14–20, 26–28, 30, 37–41, 43] of the 30 trials offer psychological withdrawal support via mobile device-based behavioral interventions either in combination with a psychological intervention delivered by a real human expert or as a primary intervention. Seven [18, 20, 26, 30, 37, 39, 41] of these seventeen trials only implement psychological withdrawal support via a mobile device-based behavioral intervention, which is neither supervised nor directed by a real human expert. These mobile device-based interventions include automated texting/messaging [30, 37, 41] (e.g., instructions on cessation, encouragement, advice, and relapse prevention messages, messages via WeChat), smartphone-delivered automated treatment [39] (i.e., an interactive smartphone-based intervention that comprises content delivered via audio/video clips), and app systems [18, 20, 26, 41] (e.g., smoking tracking, app with on-demand features, automated messaging upon Ecological Momentary Assessment; EMA responses).

Other trials offer psychological withdrawal support via a mobile device-based intervention and a psychological intervention (e.g., counseling) delivered by a real human expert. These ten trials [14–17, 19, 27, 28, 38, 40, 43] include apps designed to teach different cognitive strategies [14] (e.g., Mindfulness Training), or offer an episodic future thinking tool [27], monitor treatment success [17], enable personalized instant messaging either via WhatsApp or WeChat [43] or via local carrier [38] (e.g., tips to help quit smoking), personalized chat-based interactions [15, 40], text messages between treatment sessions to strengthen treatment success and strategies implemented in therapy sessions [19, 28], and automated, phone-based contingency management [16].

**Fig. 2:**
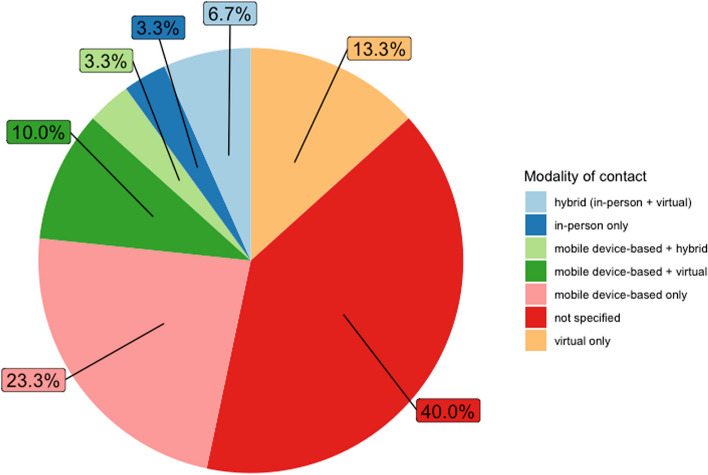
Modalities of psychological interventions. Pie chart showing the different modalities of contact employed in the psychological interventions of the clinical trials

**Fig. 3:**
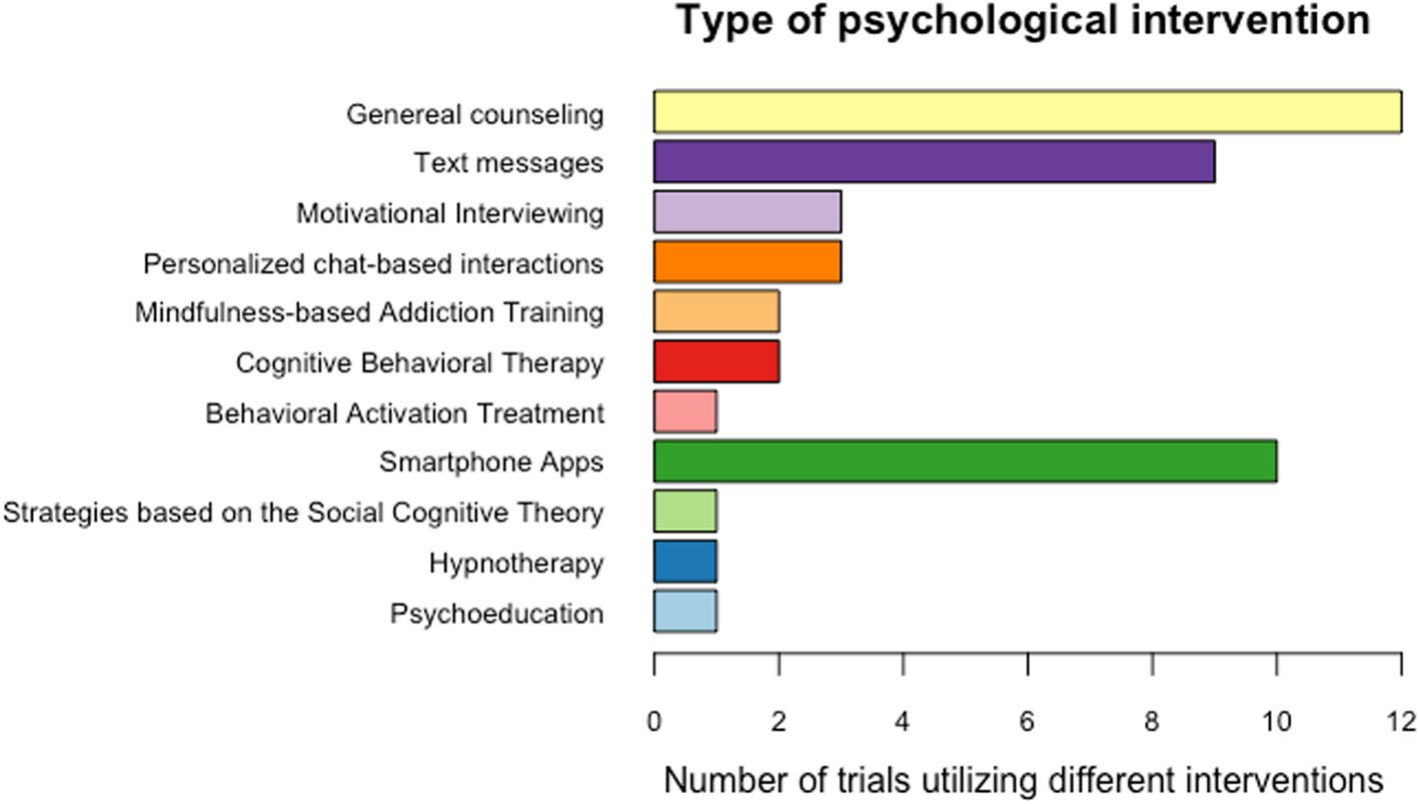
Type of psychological intervention. Graph showing the different psychotherapeutic techniques and psychological interventions employed in the clinical trials. Nicotine Replacement Therapy (NRT) and smoking cessation medication

Figure 2 summarizes the different modalities for psychological interventions and withdrawal support in the 30 trials. Conclusively, Fig. 3 visualizes the different types of psychological interventions, both mobile-device-based and delivered by a real human expert, utilized in all 30 trials.

All 30 trials implement NRTs or smoking cessation medication to accompany behavioral interventions. However, the types, durations, and dosages of NRTs and smoking cessation medications vary. Eight trials [17, 18, 20, 26, 28, 31, 40, 41] do not specify the type of NRT agent or smoking cessation medication. Nine trials only offer one type of NRT or smoking cessation medication (nicotine patches: *n* = 4 [14, 21, 25, 29], varenicline: *n* = 2 [22, 35], bupropion: *n* = 1 [42], N-acetylcysteine (NAC) [39]: *n* = 1, cystine: *n* = 1 [37]), while thirteen trials [15, 16, 19, 23, 24, 27, 30, 32–34, 36, 38, 43] offer a combination or choice of different types of NRTs or smoking cessation medications. Eleven of these trials [15, 16, 19, 24, 30, 32–34, 36, 38, 43] offer up to three of the following NRTs: nicotine patches, lozenge/gums, inhaler and/or tablets/microtabs. Additionally, two trials offer varenicline/bupropion [27] or varenicline/nicotine patches [23].

Similarly, the degree of information on the duration of NRT and smoking cessation medication varies in the trial protocols. Seventeen trials [16–18, 21, 25–28, 30, 32–34, 36, 41–43] fail to give a detailed account of the time NRT/ smoking cessation medication is offered in the intervention. Four trials [14, 15, 20, 38] offer NRT/ smoking cessation medication for six weeks or less, while eight trials offer NRTs/ smoking cessation medications for more than six weeks [19, 22–24, 29, 35, 37, 40], with two trials [31, 39] offering treatment for as long as four months or more. Furthermore, 21 [14, 16–18, 20, 21, 23, 25–31, 33, 34, 38, 40–43] of the 30 trials do not specify the dosage or number of NRTs/ smoking cessation medications provided. In five trials [15, 19, 24, 32, 36], the dosage depends upon the patients' reported baseline cigarette consumption per day or is individually monitored according to withdrawal symptoms and adjusted by a physician. Conversely, four trials [22, 35, 37, 39] report a fixed dosage in which NRT/ smoking cessation medication is administered.

#### Electronic-Nicotine-Delivery-Systems (ENDS)

Four [22, 30, 33, 37] of the 30 trials provide ENDS with varying duration, dosage, and type of ENDS. All four trials do specify the type of ENDS provided as an e-cigarette, while only one trial [37] gives detailed information on the manufacturer/brand of the e-cigarette offered. Merely one of the four trials [37] specifies the nicotine dosage in the provided ENDS in mg. Similarly, the degree of information on the duration of provided ENDS varies in the trial protocols. Two trials provide ENDS for three months [22, 37], while the other two trial protocols [30, 33] do not give any further information on the duration of provided ENDS.

### Comparison group

All studies are individually randomized and conducted in an outpatient or community setting. Ten trials include more than one experimental group.

### Outcome measures

All 30 trials measure self-reported smoking abstinence as an outcome variable of smoking cessation intervention. However, measures are taken at varying time points and with different frequencies. All 30 trials verify self-reported abstinence biochemically utilizing different techniques, while pre-define abstinence thresholds are reported in 19 of the 30 trials. Eighteen trials [16, 18, 20, 22, 23, 26–28, 30–33, 35–37, 42, 43] biochemically verify smoking abstinence by measuring expired air carbon monoxide exclusively. Meanwhile, six trials [15, 17, 21, 24, 34, 38] verify smoking abstinence utilizing saliva cotinine levels. In addition, one trial [29] verifies smoking abstinence either by expired air carbon monoxide or saliva cotinine levels or urine anabases levels, depending on when abstinence is reported. Furthermore, five trials [19, 25, 39–41] verify self-reported abstinence using expired air carbon monoxide measures or by employing saliva cotinine levels. Concerning abstinence thresholds, eleven [16, 17, 25, 26, 29, 30, 33, 35, 38–40] of the 30 trials do not define these abstinence thresholds. Six different thresholds are used (i.e., from < 4 ppm to < 10 ppm in exhaled air monoxide) in the trials measuring expired air carbon monoxide to verify abstinence. Trials using saliva cotinine levels report four pre-defined values (i.e., < 4 ng/ml to < 30 ng/ml for salivary cotinine assay).

In fifteen trials [15–19, 23, 26, 28, 29, 33, 35, 38, 39, 41, 42], a follow-up measurement occurs at six months. A longitudinal study design is implemented in ten trials [20–22, 24, 25, 27, 31, 34, 37, 40] as they plan to include follow-up measurements up to 12 months. Six trials [14, 15, 18, 25, 26, 32] take only one follow-up measurement, while eleven have at least two follow-ups [23, 24, 27, 29, 30, 35, 37, 39, 41–43]. Three or more follow-ups are taken in eleven trials [16, 17, 19, 21, 28, 31, 33, 34, 36, 38, 40], with two trials [20, 22] having as many or more than five follow-up measurements.

## Discussion

### Findings on psychotherapeutic interventions

Previous research has provided robust evidence on the effectiveness of psychotherapeutic techniques and interventions for smoking cessation, particularly when combined with pharmacological aids [7]. However, a significant challenge lies not in developing new interventions, but in improving patient engagement with existing evidence-based interventions. Of the 30 trials in our systematic review, 22 apply several psychotherapeutic methods, yet few of these address strategies for increasing treatment adherence. These findings underscore the need for further research prioritizing the barriers to treatment engagement and strategies to overcome them.

### Findings on database structure in trial registries

A major observation from analyzing the 30 RCTs is the lack of standardization in the information provided in the IRCTP and linked registries like clinicaltrials.gov. Some RCTs offer detailed descriptions of psychological interventions, while others merely mention "counseling" or the program name without further specification. Many trial protocols lack details on counseling providers or therapeutic strategies, making it difficult to draw conclusions about the suitability of psychological interventions for smoking cessation or harm reduction. This heterogeneity in trial descriptions poses challenges for researchers, funding agencies, and policymakers who rely on such data to guide future research directions. Standardizing the input of RCTs in databases like IRCTP is recommended, potentially involving a rigorous peer-review system to ensure the inclusion of necessary details. However, strict guidelines could deter trial registrations due to the increased workload. Harmonizing guidelines globally would require collaboration among experts to create universally acceptable standards. While challenging, implementing these changes could enhance research quality, advancing knowledge in this domain.

### Evaluation on harm reduction strategies

Another aspect discovered in this review relates specifically to smoking cessation and harm reduction. Only four of the 30 RCTs utilize ENDS as a harm reduction strategy, and none of the trials investigate heat-not-burn products. This underrepresentation may be due to the controversial nature of ENDS. The long-term health consequences of ENDS and their effectiveness for smoking cessation remain uncertain [44–46]. This uncertainty complicates the development of balanced medical guidelines for ENDS, despite their popularity among people who smoke [47, 48]. Opponents emphasize associated risks and high prevalence among young people, while proponents highlight the potential to reduce harmful chemical intake from tobacco. Previous studies have shown reduced toxicant exposure in exclusive ENDS users compared to conventional tobacco smokers [10, 49, 50]. ENDS likely offer better nicotine bioavailability than marketed pharmaceutical NRTs. However, concerns exist regarding toxicant levels in ENDS emissions and compensatory behavior due to lower nicotine concentrations. While ENDS reduce exposure to harmful chemicals, non-negligible levels of cardiovascular and respiratory toxicants, such as formaldehyde, acetaldehyde, and metals, remain present [10]. Opinions on ENDS's relative risks and benefits vary among scientists [51–54]. Kotz and colleagues (2022) further demonstrated the effectiveness of ENDS for smoking cessation, showing increased quit rates in individuals using ENDS compared to unassisted quit attempts and other forms of NRT [55]. Despite these promising results, the potential long-term adverse effects of prolonged ENDS use must be considered before recommending unrestricted use as a harm-reduction strategy. Our results indicate that ENDS are critically understudied, with potential underestimation of their utility as a last-option smoking cessation support while the lack of reliable longitudinal data examining long-term risks and benefits is problematic.

Furthermore, the limited number of ENDS trials likely also reflects methodological challenges. Until recently, researchers lacked access to standardized research-grade-e-cigarettes, particularly in the United States, which hindered trial design and funding opportunities. Similarly, standardization challenges exist for other types of ENDS. This situation highlights the need for improved research infrastructure and standardization of ENDS to facilitate clinical trials. Our results indicate that ENDS are critically understudied, with potential underestimation of their utility as a last-option smoking cessation support.

### Findings on geographic representation of trials

The majority of included trials are conducted in North America. This predominance may be due to greater attention to the economic burden of smoking in the US, leading to more funding for RCTs on smoking cessation and harm reduction. The influential recommendations of the US Preventive Services Task Force (USPSTF) in 2015, including statements on ENDS, may have spurred interest in this area. Such recommendations support researchers and clinicians in formulating specific research questions and RCT designs. However, to ensure global applicability, it is crucial to investigate cross-cultural differences and expand trial locations beyond Western countries. Cross-cultural research is essential to determine whether Western treatment guidelines are effective in diverse cultural contexts.

### Limitations

Several limitations should be considered in the context of this analysis and its findings. Our exclusion criteria restricted the review to ongoing RCTs following German treatment guidelines for tobacco dependence. RCTs classified as "completed," "terminated," "unknown," or "withdrawn" were excluded, potentially limiting our analysis to trials reflecting the most current knowledge. Additionally, focusing on German guidelines may not fully capture international guidelines, which can vary by country due to cultural, healthcare, and resource differences. However, the German guidelines closely align with WHO guidelines, offering a standardized benchmark to evaluate ongoing RCTs. Our evaluation of international RCTs was conducted based on globally recognized practices, enhancing the review's generalizability and consistency.

The IRCTP database updates at varying frequencies from linked trial registries, so the information available at the time of our search may not include all currently registered trials. The authors are involved in an ongoing smoking cessation RCT registered in the German Clinical Trials Register (DRKS), which was not yet available on the IRCTP at the time of our search. Therefore, information from other registries may also have been omitted due to delayed updates.

## Conclusion

This systematic review underscores the ongoing efforts to address smoking cessation and harm reduction through randomized controlled trials (RCTs), particularly highlighting a significant concentration of research in North America. The findings reveal a notable focus on socioeconomically disadvantaged and uninsured populations, but a gap in addressing cancer survivors and individuals with smoking-related diseases. The variability in biochemical verification techniques and thresholds for abstinence, along with inconsistent reporting of NRT and medication details, points to a need for greater standardization in trial methodologies.

The review also identifies a diverse application of psychological counseling methods, with many trials employing motivational interviewing and other established frameworks. While existing psychotherapeutic methods have demonstrated efficacy, the larger challenge lies in improving patient engagement with these treatments rather than developing new interventions, which suggests a need for implementation research. Furthermore, the under-studied nature of ENDS remains a critical issue, compounded by the lack of standardized, research-grade products. Expanding research beyond North America and standardizing trial reporting could significantly enhance the global relevance and applicability of smoking cessation strategies.

## Supplementary Information

Supplementary Material 1.

## Data Availability

No datasets were generated or analysed during the current study.

## Abbreviations

GDP: Gross Domestic Product
COPD: Chronic obstructive pulmonary disease
MI: Motivational Interviewing
CBT: Cognitive-behavioral therapy
RCTs: Randomized Controlled Trials
NRT: Nicotine Replacement Therapy
ENDS: Electronic Nicotine Delivery Systems
WHO: World Health Organization
FCTC: Framework Convention on Tobacco Control
S3: (Refers to the German S3 guidelines for tobacco dependence)
IRCTP: International Registry of Clinical Trials Platform
HIV: Human Immunodeficiency Virus
PTSD: Post-Traumatic Stress Disorder
MAPS: Managed Problem Solving
EMA: Ecological Momentary Assessment
USPSTF: United States Preventive Services Task Force
PAD: Peripheral Arterial Disease
DRKS: German Clinical Trials Register
OR: Odds Ratio
RR: Relative Risk
