# Supplementary material for: Smoking cessation and harm reduction: a systematic overview of ongoing, randomized controlled trials
Source: BMC Psychiatry. 2024 Dec 17;24:897. doi: 10.1186/s12888-024-06342-6 (PMC11650832; doi:10.1186/s12888-024-06342-6)
Supplement: Supplementary file 1 — Supplementary Material 1. [file 12888_2024_6342_MOESM1_ESM.docx]

**Supplement**

**Search strategy**

DATABASE SEARCH – March, 2023

Database: International Clinical Trials Registry Platform (ICTRP) 1990 January 1 to 2023 March

Search Strategy:

| Search terms | Results |
| --- | --- |
| smoking cessation OR smoking intervention OR harm reduction OR switching trial AND RCT AND interventional  Status: Recruiting | 282 records for 282 trials |

**Table 1. Overview of the 30 RCTs meeting all criteria according to our configuration of the PICOS-scheme**

| Responsible party | Setting/trial location | Population | Intervention components | Dosing of intervention | Measurement of smoking cessation |
| --- | --- | --- | --- | --- | --- |
| Institut Cancerologie de l'Ouest(1) | France | smokers with cancer requiring surgical treatment | control group: active comparator; experimental groups: Motivational Interviewing/Motivational Interviewing + Cognitive Behavioural Therapy/ Motivational Interviewing + Hypnotherapy | Motivational Interviewing (Y); Cognitive Behavioural Therapy (Y/N); Hypnotherapy (Y/N): 6 months of NRT (type of NRT, duration, and dosage not specified) | Self-report; exhaled CO |
| Boston Medical Center(2) | United States,  China | patients with HIV who smoke regularly | control group: self-help guide and NRT; experimental group: 8 week quit smoking program intervention | counseling sessions: 4 sessions over a course of 8 weeks (decreasing duration); cell phone messages: brief messages with tapering frequency; self-help guide for quitting smoking; NRT: transdermal patches or gums (duration and dosage not specified) | Self-report; exhaled CO |
| Haukeland University Hospital(3) | Norway | patients with severe opioid dependence receiving OAT who smoke regularly | control group: initial screening only; experimental group: smoking cessation counseling + prescription-free NRTs | behavioral counseling sessions: once a week; NRT: patches, gum and lozenges, dosage individually chosen (duration not specified) | Self-report; exhaled CO |
| University of Miami(4) | United States | cancer survivors who smoke regularly | control group: initial screening only; experimental group: Craving-to-Quit app or In-person Mindfulness Training | orientation session in Mindfulness Training: 1 session (90 mins); brief follow-up calls one day before the quit date, at the end of treatment, and at 3-month follow-up (15 mins each); Craving-to-Quit app: compromised of 22 modules for 22 days (5-15 minutes each); group Mindfulness Training sessions: total of 8 sessions (45-60 minutes each); NRT: 6 weeks of transdermal patches are provided (duration and dosage not specified) | Self-report; exhaled CO |
| University of Wisconsin, Madison(5) | United States | low-education and uninsured individuals who smoke regularly | control group: not specified; experimental groups: four-factor fully crossed factorial design | counseling: 4 proactive calls (20 mins each) or 1 proactive call (20 mins); financial incentives for treatment engagement (Y/N); text messaging in support of cessation: 6-8 weeks of text messages (up to 5 messages per day) (Y/N); NRT: 2 weeks of transdermal patches or 4 weeks of lozenges and transdermal patches are provided (dosing dependent upon reported baseline cigarettes per day) | Self-report; saliva cotinine levels |
| University of Oklahoma(6) | United States | socioeconomically disadvantaged smokers | control group: standard care; experimental group: Contingency Management | Automated Mobile Contingency Management: financial incentives contingent upon smoking abstinence; telephone counseling; NRT: transdermal patches and gum/lozenges (duration and dosage not specified) | Self-report; exhaled CO |
| University of California, San Francisco(7) | United States | young Veterans with PTSD who smoke regularly | control group: active comparator; experimental group: tech-facilitated integrated care (IC) intervention | Integrated Care (IC) treatment protocol for smoking cessation (duration and dosage not specified), Stay Quit Coach (SQC) smart phone application, coVita Bedfont® Scientific Ltd iCO® Smokerlyzer® device and application; NRT: (type, duration and dosage not specified) | Self-report; saliva cotinine levels |
| University of Oklahoma(8) | United States | regular smokers | control group: active comparator; experimental group: smart treatment app | Smart treatment app: EMA delivery and data transfer system, automated messages based upon EMA responses, and on-demand content; NRT: (type, duration and dosage not specified) | Self-report; exhaled CO |
| Société de Formation Thérapeutique du Généraliste Recherche(9) | France | smokers with low socio-economic position | control group: active comparator/standard care; experimental group: The STOP intervention | The STOP intervention: health professional led intervention, routine care and adapted advice (duration and dosage not specified); NRT: transdermal patches, inhalers, gum, tablets, etc. (duration and dosage not specified); ENDS: electronic cigarette + e-liquid | Self-report; exhaled CO |
| Georgia State University(10) | United States | regular smokers | control group: usual care; experimental groups: iQuit Mindfully or Mindfulness-based Addiction Treatment or iQuit Mindfully + Mindfulness-based Addiction Treatment | Mindfulness-based Addiction Treatment: 8 weekly group sessions (2 hours each); Self-help materials; iQuit Mindfully: text messages on each day between treatment sessions; NRT: 8 weeks of transdermal patches or lozenges are provided (dosing dependent upon reported baseline cigarettes per day) | Self-report; exhaled CO |
| University of Houston(11) | United States | African American smokers | control group: QuitGuide app + NRT; experimental group: Mobile Anxiety Sensitivity Program for Smoking (MAPS) app + NRT | Mobile Anxiety Sensitivity Program for Smoking (MAPS) app: educational videos, tailored messages, and interoceptive exercises designed to help the user overcome negative feelings of stress and nicotine withdrawal, framed within the cultural context of interoceptive stress among African American smokers, utilizes Ecological Momentary Assessments (EMAs); NRT: (type, duration and dosage not specified) | Self-report; exhaled CO |
| University of Twente(12) | Netherlands | Smokers with COPD | control group: brief self-help intervention; experimental group: reduction-to-quit smoking intervention | Behavioral counseling: eight small-group sessions and four telephone contacts tailored messages; NRT: transdermal patches and/or gum and/or tablets (duration and dosage not specified) | Self-report; saliva cotinine levels |
| University of South Florida(13) | United States | smokers living with certain chronic conditions | control group: sham comparator; experimental group: Positively Me | Positively Me Intervention: 12 sessions; NRT: offer of transdermal patches, but not part of the experimental design | Self-report; saliva cotinine levels |
| Heart Foundation, Australia(14) | Australia | smokers scheduled for elective surgery | control group: usual care; experimental group: behavioral/drug treatment | Call back service for telephone support (Quitline); NRT: lozenges and transdermal patches (for heavier smokers), dose and duration of NRT treatment will vary according to nicotine dependence | Self-report; exhaled CO |
| Sir Mortimer B. Davis - Jewish General Hospital(15) | Canada | smokers diagnosed with Acute Coronary Syndrome requiring hospitalization | control group: active comparator: combination therapy arm; experimental group: Varenicline + counseling | counseling: no information on content, frequency or duration; NRT: Varenicline with increasing dosage for 12 weeks; ENDS: e-cigarettes and cartridges/pods (no information on nicotine concentration given) for 12 weeks (Y/N) | Self-report; exhaled CO |
| University of Pennsylvania(16) | United States | patients with HIV who smoke regularly | control group: Varenicline + standard cessation counseling; experimental group: Nicotine metabolite ratio-tailored medication + standard cessation counseling + Managed Problem-Solving adherence intervention | Managed Problem Solving (MAPS) adherence intervention: up to 5 sessions over 8 weeks; Standard behavioral smoking cessation treatment: up to 5 therapy sessions over 8 weeks; NRT: 12 weeks of transdermal patches or varenicline are provided (dosage not specified) | Self-report; exhaled CO |
| NYU Langone Health(17) | United States | low-income smokers | control group: active comparator: standard smoking cessation coaching; experimental group: integrated Financial-Smoking Cessation Counseling | counseling: 5 financial counseling sessions over approximately 8 weeks; financial incentives; NRT: lozenges, gums and transdermal patches (provided for 8 weeks with decreasing dosage) | Self-report; saliva cotinine levels |
| Hennepin Healthcare Research Institute(18) | United States | patients with an acute coronary syndrome diagnosis within the past 30 days who smoke regularly | control group: Smoking Cessation and Health & Wellness; experimental group: Behavioral Activation Treatment for Cardiac Smokers | Behavioral Activation Treatment: 5 counseling sessions (first session 1 hour, last 4 sessions 30 minutes each); NRT: offered transdermal patches (duration and dosage not specified) | Self-report; saliva cotinine levels or exhaled CO |
| University of Oklahoma(19) | United States | regular smokers | control group: active comparator: The Standard Helpline Care (SC) group; experimental group: OKquit group | OKquit Smartphone App: daily check-ins and weekly surveys, Ecological Momentary Assessments (EMAs) for 27 weeks, tailored smoking cessation messages, on-demand smoking cessation content + Standard Oklahoma Helpline care; NRT: (type, duration and dosage not specified) | Self-report; exhaled CO |
| University of Pennsylvania(20) | United States | Black, Hispanic, and/or have low socioeconomic status or rural residence who smoke regularly and are referred to lung cancer screening | control group: basic usual care; experimental groups: enhanced usual care; enhanced usual care + financial incentives; enhanced usual care + financial incentives + mobile health application | Ask-Advise-Refer (AAR): provide informational resources such as hotlines, specialized clinics, or smoking cessation classes; financial incentives for biochemically-confirmed, sustained abstinence for 6 months (Y/N); mobile health application (Y/N);  NRT: varenicline or bupropion (duration and dosage not specified), (other types of NRT mentioned but not specified, duration and dosage not specified) | Self-report; biochemical confirmation |
| University of Auckland, New Zealand(21) | New Zealand | regular smokers | control groups: active comparator monotherapy (cytisine only), monotherapy (nicotine e-cigarette only); experimental group: combination therapy (cytisine plus a nicotine e-cigarette) | text-based smoking cessation support: offered for 6 months; NRT: cytisine (standard dosing); ENDS: e-cigarette (Nicotine concentration 30mg/ml (3%), tobacco flavor) provided for 12 weeks | Self-report; exhaled CO |
| Baystate Medical Center(22) | United States | patients admitted with a cardiac or pulmonary disease diagnosis who smoke | control group: Enhanced usual care (EUC) (active comparator); experimental groups: personalized care: nurse practitioner led Tobacco Treatment Team (NPT3) | multi-component, multi-disciplinary smoking cessation intervention (Y/N): personalized advice, education, and coaching provided by a Tobacco Treatment Counselor + smoking cessation text messaging program designed to maintain motivation, encourage medication adherence, and allow communication with the treatment providers (no further information on frequency and intensity); NRT: sample ad-lib (duration not specified) | Self-report; exhaled CO |
| The University of Hong Kong(23) | Hong Kong | regular smokers | control group: Placebo (Regular text-based support and nicotine replacement therapy sampling); experimental group: personalized chat-based support and nicotine replacement therapy sampling | psychoeducation; personalized chat-based interactions (duration of 3 months); brief follow-up calls: regularly (15-30 mins); NRT: 8 weeks of treatment (type and dosage not specified) | Self-report; saliva cotinine levels and exhaled CO |

**Table 2. Detailed summary of components for psychological smoking cessation interventions delivered by a real human expert**

| Type of intervention | Content | Frequency of contact | Intensity | Modality of contact | Type of provider |
| --- | --- | --- | --- | --- | --- |
| general counseling (*n* = 12(2,3,5–7,12,14,15,17,20,22,24,25)) | asked about progress in cessation attempt, cigarettes smoked the last week are recorded. Goal is set for the next week. Information on typical nicotine withdrawal symptoms and ameliorating techniques (*n* =1(3)); financial incentives for treatment engagement (*n* =1(5)); financial incentives contingent upon biochemically-verified smoking abstinence (*n* = 2(6,20)); using a standard, evidence-based protocol (*n* = 1(5)); content is consistent with that delivered in the integrated-care treatment protocol for veteran smokers with post-traumatic stress disorder (*n* =1(7));  financial smoking cessation coaching (*n* = 1(17)); tailored behavioral intervention for treating tobacco dependence (*n* = 1(24)); Ask-Advise-Refer: ask smokers about their desire to quit smoking, advise them to quit, and provide informational resources (*n* = 1(20)); not specified (*n* = 4(2,12,14,15)) | Frequency specified (weekly) (*n* =2(3,6)); contingency (1-4 sessions (5); 8 sessions(7,24);  8 group sessions + 4 individual sessions(12); 5 sessions over 8 weeks(17); 4 sessions over 8 weeks(2); timeframe specified (12 weeks) (*n* = 1(25)); not specified (*n* = 4(14,15,20,22)) | contingency (15-20 minutes(2,5); 20-60 minutes(7)); not specified (*n* = 10(3,6,12,14,15,17,20,22,24,25)) | telephone calls (*n* = 3 (5,6,14)); telephone calls/videoconferencing (*n* = 1(7) );  hybrid (in-person and telephone calls) *n* = 1(12); not specified (*n* = 8(2,3,15,17,20,22,24,25)) | tobacco treatment counseling (*n* = 1(22)); pulmonary nurses (*n* = 1(12)); clinician (*n* = 1(20)); not specified (*n* = 10(2,3,5–7,14,15,17,24,25)) |
| psychoeducation (*n* = 1(23)) | Health talk provided information about hazards of tobacco (active smoking, second- and third-hand smoke), benefits of quitting smoking and methods to quit smoking(23) | Not specified(23) | not specified(23) | not specified(23) | not specified(23) |
| Motivational interviewing (*n* = 3(1,9,26)) | not specified (*n* = 3(1,9,26)) | contingency (1-3 sessions) (*n* =1(1)); Frequency specified  (weekly) (*n* =1(26)); not specified (*n* =1(9)) | not specified (*n* = 3 (1,9,26)) | not specified (*n* = 2 (1,9)); telephone calls (*n* = 1(26)) | psychotherapist (*n* = 1(1)); medical doctor (*n* = 1(9)); Motivational Interviewer (*n* = 1(26)) |
| Mindfulness-(based addiction) training (*n* = 2(4,10)) | the overarching theme of momentary awareness and acceptance of cravings and affect (e.g., stress, anxiety etc.) will be introduced and reinforced in complementary ways throughout the training(4); teaching mindfulness and cognitive-behavioral strategies for smoking cessation(10) | contingency (8 sessions, twice a week for 4 weeks(4); 8 weekly sessions(10)) | 45-60 minutes(4); 2 hours(10) | in-person(4); virtual(10) | instructors experienced in Mindfulness Training (MT) (a single therapist with >4 years of training in MT)(4); not specified(10) |
| Cognitive Behavioral Therapy (*n* = 2(1,27)) | not specified(1); active sessions, with treatment components practiced within and outside of the session(27) | contingency (6 sessions (1); 9 sessions delivered over 10 weeks in weekly intervals(27)) | not specified(1); 60 minutes(27) | not specified (*n* = 2(1,27)) | psychotherapist(1); not specified(27) |
| Hypnotherapy (1) | not specified (1) | contingency (3 sessions) (1) | not specified (1) | not specified (1) | hypnotherapist(1) |
| Managed Problem Solving (MAPS) adherence intervention (*n* = 1(16)) | therapeutic process that involves the systematic delineation of a participant's medication adherence problems and construction of a series of individualized solutions that therapists and participants explore together(16) | contingency (5 sessions, delivered over 8 weeks)(16) | not specified(16) | hybrid (in-person and telephone calls)(16) | not specified(16) |
| Behavioral Activation (BA) treatment (*n* = 1(18)) | integrating gold standard smoking cessation counseling with existing BA based mood management techniques(18) | contingency (5 sessions, delivered over 12 weeks) (18) | 30 mins – 1 hour (18) | hybrid (in-person and telephone calls) (18) | not specified (18) |
| strategies based on the Social Cognitive Theory (*n* = 1(13)) | promote smoking cessation in people with certain health conditions(13) | contingency (12 sessions)(13) | 1.5 hours(13) | video conferencing(13) | not specified(13) |

**REFERENCES**

1. Institut Cancerologie de l’Ouest. Evaluation of Different Smoking Cessation Protocols: Nicotine Replacement, Motivational Interviewing (MI), Cognitive Behavioural Therapy (CBT) or Hypnotherapy in Cancer Patients Scheduled for Surgery [Internet]. 2021. Available from: https://clinicaltrials.gov/show/NCT04899492

2. Boston Medical Center. Quit For Life (QFL): Smoking Cessation Among Chinese Smokers Living With HIV [Internet]. 2021. Available from: https://clinicaltrials.gov/show/NCT05020899

3. Haukeland University Hospital. Easy Access to Smoking Cessation for People Receiving Opioid Agonist Therapy Who Are Smoking Tobacco [Internet]. 2022. Available from: https://clinicaltrials.gov/show/NCT05290025

4. University of Miami. Mindfulness Based Smoking Cessation Among Cancer Survivors [Internet]. 2019. Available from: https://clinicaltrials.gov/show/NCT04038255

5. University of Wisconsin. Improving Quitline Support Study [Internet]. 2018. Available from: https://clinicaltrials.gov/show/NCT03538938

6. University of Oklahoma. Mobile Contingency Management for Smoking Cessation [Internet]. 2021. Available from: https://clinicaltrials.gov/show/NCT04881630

7. University of California San Francisco. Tech and Telephone Smoking Cessation Treatment for Young Veterans With PTSD [Internet]. 2018. Available from: https://clinicaltrials.gov/show/NCT03552978

8. University of Oklahoma. Smartphone Based Smoking Cessation Intervention [Internet]. 2018. Available from: https://clinicaltrials.gov/show/NCT03740490

9. Société de Formation Thérapeutique du Généraliste Recherche. Preference-based Tools for Smoking Cessation Among Disadvantaged Smokers, a Pragmatic Randomised Controlled Trial [Internet]. 2020. Available from: https://clinicaltrials.gov/show/NCT04654585

10. Georgia State University. Mindfulness-based Smoking Cessation Enhanced With Mobile Technology [Internet]. 2021. Available from: https://clinicaltrials.gov/show/NCT04965181

11. University of Houston. Smoking, Stress, and Mobile Technology [Internet]. 2021. Available from: https://clinicaltrials.gov/show/NCT04838236

12. University of Twente. Effects of a reduction-to-quit smoking programme in patients with COPD: the REDUQ study. [Internet]. 2010. Available from: https://trialregister.nl/trial/2110

13. University of South Florida. The Positively Quit Trial for Smoking Cessation [Internet]. 2020. Available from: https://clinicaltrials.gov/show/NCT04449458

14. Frankston Hospital. Stop for the Op and stop for life; Can smoking cessation be increased before elective-surgery by an offer of free mailed nicotine replacement therapy (NRT) and Quitline counselling to smokers waiting for surgery? [Internet]. 2019. Available from: https://anzctr.org.au/ACTRN12619000032156.aspx

15. Sir Mortimer B. Davis - Jewish General Hospital. Aggressive Smoking Cessation Trial (ASAP) [Internet]. 2022. Available from: https://clinicaltrials.gov/show/NCT05257629

16. University of Pennsylvania. Optimizing Tobacco Use Treatment for PLWHA [Internet]. 2019. Available from: https://clinicaltrials.gov/show/NCT04176172

17. NYU Langone Health. Integrating Financial Coaching and Smoking Cessation Coaching [Internet]. 2021. Available from: https://clinicaltrials.gov/show/NCT05154669

18. Hennepin Healthcare Research Institute. Post Acute Cardiac Event Smoking (PACES) Study [Internet]. 2018. Available from: https://clinicaltrials.gov/show/NCT03413423

19. University of Oklahoma. Testing a Novel Smartphone Application for Smoking Cessation With the Oklahoma Helpline [Internet]. 2022. Available from: https://clinicaltrials.gov/show/NCT05539209

20. University of Pennsylvania. Comparing Smoking Cessation Interventions Among Underserved Patients Referred for Lung Cancer Screening [Internet]. 2021. Available from: https://clinicaltrials.gov/show/NCT04798664

21. University of Auckland NZ. Cytisine and E-cigarettes With Supportive Text-messaging for Smoking Cessation (Cess@Tion) [Internet]. 2021. Available from: https://clinicaltrials.gov/show/NCT05311085

22. Baystate Medical Center. Smoking Cessation Pharmacotherapy-Nurse Practitioner Led Tobacco Treatment Team Study [Internet]. 2023. Available from: https://clinicaltrials.gov/show/NCT05733767

23. The University of Hong Kong. Smoking Cessation Programme in Workplaces in Hong Kong (Phase ?) [Internet]. 2021. Available from: https://clinicaltrials.gov/show/NCT04772521

24. University of Maryland. Smoking Cessation Interventions for People Living With HIV in Nairobi, Kenya [Internet]. 2017. Available from: https://clinicaltrials.gov/show/NCT03342027

25. Maastricht University. Helping more smokers to quit by combining varenicline with counselling for smoking cessation. The COVACO randomized controlled trial. - COVACO [Internet]. 2009. Available from: https://www.clinicaltrialsregister.eu/ctr-search/search?query=eudract_number:2009-016446-50

26. University of Western Australia. Using Admissions to a “Smokefree” hospital to promote cessation of smoking in mental health inpatients versus a representation of the general population. [Internet]. 2009. Available from: https://anzctr.org.au/ACTRN12609000627257.aspx

27. Massachusetts General Hospital. Effectiveness of an Integrated Treatment to Address Smoking Cessation and Anxiety/ Depression in People Living With HIV [Internet]. 2019. Available from: https://clinicaltrials.gov/show/NCT03904186
